# Re-thinking knowledge landscapes in the context of Grounded Aboriginal Theory and online health communication

**DOI:** 10.3325/cmj.2018.59.33

**Published:** 2018-02

**Authors:** Kishan Kariippanon, Kate Senior

**Affiliations:** 1 University of Wollongong, School of Health and Society, Faculty of Social Science, Australia *kishan@uow.edu.au*; 2 University of Wollonong, School of Health and Society, Australia

The Aboriginal people of North East Arnhem Land, Australia, are a diverse community speaking several languages, but united through a kinship system connecting individuals and clans between two moieties of the Yolngu nation: the Yirritja and Dhuwa (1,2). Every animate or inanimate object belongs to either Yirritja or Dhuwa moiety, and both moieties complement each other in the Yolngu structure. The history of this Indigenous nation has been one of survival and resilience, especially during the battle for control of their ancestral lands and maritime borders that culminated in the Land Rights Act of the Northern Territory 1977 (3). The Yolngu nation has a history of engaging and negotiating knowledge landscapes of the ‘Other’, ie, Macassan traders and Christian Missionaries (3-6), and their ability to combine traditional and contemporary methods of communication is embellished in the Yirrkala Bark Petition to the Australian Parliament to recognize the ownership of their land (3,6). Here the relatively new technology of the cold, heavy, steel typewriter was incorporated with the traditional bark painting to convey complex and intricate stories of creation, ceremony and law.

Despite the success of the Land Rights movement of the seventies, Aboriginal communities in the Northern Territory, were invaded in 2007 by the Commonwealth Government under the premise of addressing alleged child abuse (7). This dramatic reaction on the part of the Government was even called the Northern Territory Emergency Intervention and took place amidst a lengthy history of the decline of Aboriginal health in the region (8). The general health and well-being of Aboriginal people in the Northern Territory “retained low life expectancies, especially for men, and high levels of avoidable mortality” (9). In one particular town, researchers found the death rate at 4.5 times higher than the general Australian population (9). Health inequalities between Indigenous and non-Indigenous populations were in stark contrast, with high rates of infant malnutrition, parasitic diseases and respiratory problems in the former (10).

The proliferation of mobile phones and social media into remote settings, such as North East Arnhem Land, was possible as a result of telecommunication network provider Telstra’s increased reach of the 3G mobile network with Internet capability (11-14). It may also have been the result of population growth in non-Indigenous residents working in mining towns across the Northern Territory, as a result of the high demand for Australia’s natural resources for export. Aboriginal people, as a consequence of both the interaction with non-Indigenous communities and their demand for mobile network connectivity, along with the availability of mobile phones for purchase in local post office or shops, have rapidly adopted the relatively affordable and easy to use technology for their benefit. The functionality of mobile phones and social media allows Aboriginal communities and families to overcome their lack of economic resources required to travel across vast distances for the maintenance of traditional law, ceremony, and the reinforcement of kinship relations (13,14).

Within the nexus of globalisation and an Aboriginal modernity in its early stages, health promoting and social marketing messages have taken on new technology and populated social media and mobile phones with Facebook posts and SMS text messages.

The need to communicate is also an “essential tool for establishing good psychosocial conditions for the user, the professionals, and people in general” (15) and has been observed to be practiced in Yolngu communities with internet capable mobile networks as natural phenomena. The technology that is available in the remote community of North East Arnhem Land enabled video material to be shared via video calls, posted and viewed online, or even shared over Bluetooth connectivity between mobile phones in close proximity. This has also created a set of new health promotion opportunities for health services and for collateral communication channels between health services, the individual, and the community to be explored and subsequently forced upon the community. One of the advantages of such a method of engagement is the lower cost, requiring less human resources but measureable outputs as opposed to the ‘sittin tellin story’ – a process of generating complex narratives by combining different perspectives through collective reflexivity, story-telling, and the use of metaphors – a method that Senior (16) will draw upon in the analysis section.

The intention to create a dialogue with people from the target community is often forgotten by public health practitioners in the process of using social media and mobile phones. Online discussions are discreetly feared by health service providers, as they may provide an uncensored avenue for critique, which may negatively impact on the organization’s reputation or compromise patient confidentiality. We strongly agree and support the use of new technology as it is laden with potential to “enable the flow and exchange of knowledge” (17). However, from this study, we learnt that in the Aboriginal context, there are few opportunities for meaningful exchange between the stakeholders in technology based interactions. The European philosophy of theory and practice are seen as two separate entities, but the opposite is true according to Grounded Aboriginal Theory, where theory and practice are interwoven (18). Grounded Aboriginal Theory describes a ‘ground-up’ approach to knowledge generation through an ethnographic method, where discourse between the Indigenous and non-Indigenous knowledge landscapes are stimulated not steered or influenced upon by set objectives or outputs. It is this separation of theory and practice in mainstream Australian health communication that runs counterproductive to the groundedness of Aboriginal Theory (16,19).

Is it possible that the widespread acceptance and appropriation of social media and mobile technology has displaced the central importance of content design? Content design is not just based on theory, but on practice as well, especially through the use of video and visual ethnography for social media social marketing. Has the search for ‘viral marketing’ and ‘Likes’ taken the place of a process oriented toward negotiation and the translation of research between two separate knowledge landscapes (20)? Western concepts of behaviors and the process of health education takes little account of traditional Aboriginal methods of negotiation and knowledge creation where health messages require reflexivity (21) and negotiation between the health services, the individual and the community (16,19,20).

According to Nakata (22), the Indigenous epistemological basis of knowledge construction is embedded in a variety of ways such as “storytelling, memory making, narrative art and performance; in cultural and social practices, of relating to kin, of socialising children in ways of thinking, [and] of transmitting knowledge”. The ability of Aboriginal people to understand Western epistemology through colonisation, the Christianisation of their lived experiences, and the forced use of the English language did not destroy the Aboriginal lens in which their worldview is shaped (23). Today, the consumption of popular culture and emergent technology is still based on the Aboriginal knowledge landscape and its “groundedness” was even more evident during the process of ethnographic filmmaking (24) for an anti-tobacco social marketing campaign produced by the researchers in collaboration with Aboriginal social marketers and senior elders from a regional health service provider (20).

As a result of lessons learnt post-production of a digital media anti-tobacco campaign (20), we focused on the analysis of health services that formulate, broadcast, and dictate what healthy behavior is through the use of social media and mobile phones. We argue that Aboriginal health services are unfairly bounded by the western biomedical model, lacking in sufficient and overt consideration of the traditional processes of knowledge creation where Aboriginal people and their ways of knowing is acknowledged and embraced without prejudice.

## Integration of mobile technology into peoples’ knowledge landscapes

The ethnographic study of the social life of mobile phones and social media in a remote Indigenous community conducted between 2012 and 2015 demonstrated the level of integration of mobile technology and social media by young people in a remote Aboriginal community (25). These technologies are often perceived as novel by the non-Indigenous outsider, but our informants told us that such technology was simply used for everyday purposes. The Indigenous youth have turned to mobile technology and social media to communicate across vast distances, ie, between remote communities in the region where access by road is cut off during monsoonal rains, to practice culture, and to reinforce relationships.

A young Aboriginal man in in his early thirties commented on the pros of communication between kin on social media and mobile phones. He was engaged in a video call, broadcasting a funeral ceremony to a family member who was unable to make the journey. He said that community members were “able to ring family and friends, or use digital technology for phone banking, to [make] video calls, live stream teaching, friends, students, Government people, sports, culture, leisure and many more from place to place, out bush to city, country to country, and more”. The use of the technology clearly aligned itself naturally to the needs of a community separated by vast distances. The mobile phone had become the new ‘message stick’. But the novelty of this medium of communication is eclipsed by the primary practice of sharing and inclusivity in the observance of traditional funeral ceremonies.

The excitement of mobile technology and social media had prompted many elders, young adults, and especially teenagers to see it as an organic element of their lived experience instead of a novel piece of technology. The ability to crowdsource solutions and share resources had become more convenient since the adoption of digital communication and mobile phones rather than creating a new set of practices of founding a new method of communication. A public message on social media was posted asking for a specific product available for sale in only one community. The status update said “to all my Community FaceBook friends can any of yous tell me where they sell Cool C please i really need to buy them now before i go back to Town tomorrow?” This explains the practicality of the use of technology in everyday lives where the focus is still on reinforcing Indigenous values instead of an exploration on new ways of being.

The next example is where a man in his thirties decided to discuss and crowdsource ways of quitting smoking and specifically asked for options outside the biomedical approach. He wrote: “Any tips to quit smoking without the nicotine patch? Help me please friends”. From this example we learnt that it is possible that Aboriginal methods or knowledge systems are considered and sought after online, and could be more engaging, particularly in smoking cessation activities. The ordinariness of the mobile phone now integrated into everyday life is possibly no longer a novelty. This idea stands in opposition to the ‘special powers’ attributed to mobile technology as an intermediary by public health and health promotion programs (14). We observed that there is a paucity of health programs that engage with Indigenous peoples’ knowledge landscapes in this community where the health promoting messages becomes an end point of discussion instead of a facilitator of discourse.

Cindy, a mother and community development worker talked about the recently completed anti-tobacco social media marketing campaign and voiced her opinion on the expectations of such behavior change strategies that used social media and mobile phones. She said: “It think the people heard the message but didn’t really take it in. So yeah, if there was another health promotion, I would want [it] to be catchy like that with good choreography and dancing, a lot of young kids involved and they usually have the best ideas and it attracts attention in the communities”. From her comments, it is clear to us that the presentation of ideas using social media was found to be entertaining and participatory, but it may have missed an opportunity to utilize traditional methods of reflection and discourse.

These messages excluded the fact that tobacco has been in Yolngu culture for many centuries and was part of the kinship system and funeral ceremony. It is used to remember those who have passed on (20,26). We observed that when traditional practices with a sacred item like tobacco were excluded from the digital social marketing anti-tobacco content, any further discourse was prematurely precluded. For example, Cindy said that she had often heard comments about the online video from her family and networks during the campaign: “that’s a funny clip, are they gonna be doing any more?” However, she clearly recollected that she hadn’t “heard much comment about people wanting to stop smoking”. According to our informants, the health promoting messages do not engage with Indigenous ways of telling, of transferring knowledge or creating a complex plot or metaphor and instead promotes simplistic ideas of causation in regards to illness and well-being as a result of smoking tobacco. In this context, social media as a tool for communication may only catch people’s attention briefly, and possibly, superficially, while assuming that the message itself will be perceived as the ‘truth’ an accepted without negotiation.

An Aboriginal senior elder, Jan, who was a mother of teenage children, acknowledged the community’s input in the design of social media messages as somewhat didactic and had limited impact. On the high prevalence of smoking in her community especially among teenagers and young adults, and the effect of the anti-tobacco campaign, she said, that it would “take a lot more things to [create] change. More innovative stuff. Maybe [the videos] were effective, but it’s an issue that’s not gonna be solved by sending one video around”.

Jenny, a senior woman from the community development sector who has worked closely with teenage girls to promote healthy eating, reflected on how her participants interacted with the process of producing and engaging with the content of a healthy eating video. Even though her program participants were engaged as partners and were in control of creating health messages for their peers using social media and mobile phones, she was ultimately unsure of its effectiveness and its translation from the western knowledge landscape to the Aboriginal. She said: “whether they actually took it on as information for healthy food, I don’t know”, but she was certain that the process of production was highly beneficial to building capacity and self-esteem.

Considering Jenny’s comments, we may extrapolate from the theory according to Christie (19) that young people were not so much interested in the “content of their knowledge, but the shared background which makes truth claims and performances possible and assessable”. We consider this observation to further open the question of whether the use of social media and mobile phones in promoting health messages can be designed to be more effective by drawing on the Groundedness of Aboriginal Theory through a process of reflexivity, ‘sittin telling story’, and performance, instead of a focus on the causality of mortality and morbidity associated with lifestyle choices.

## Analysis

The production of social media messages, text messages, and video to engage Aboriginal community members, particularly teenagers and young adults, are often presumed by health professionals and social marketers prima facie as effective translational tools. These tools artificially assist the health services to bridge the ideological separation between the western biomedical model and Grounded Aboriginal Theory to create a neutral knowledge landscape as a compromise. According to Gajovic and Svalastog (27), knowledge is not absolute and therefore even the western biomedical model is challenged by its constantly changing relevance and is constantly re-evaluated.

Morphy (6) explains that the traditional method of communication from an Aboriginal theoretical perspective draws on the complexity of the process. A relevant example is the process of making bark paintings (6). The bark painting is composed of distinct features, from natural elements, organized and executed in a particular order. “There is a base colour, then various internal subdivisions, figurative representation, geometric background patterns and cross-hatching” is added to the work. The process is transformative rather than a linear step by step procedure reduced into simplistic arithmetic formulae.

It begins from a rough sketch, taking up not more than an hour. The subsequent cross hatching that takes place, however, overlaps the outlines of the rough sketch. In time, the successful cross hatching painting attains a “shimmering brilliance and in addition to its separate components, and become clearly defined” (6).

This process of making the dull bright in bark paintings is translated into the process of knowledge transmission as making something blurry, clarified, and discernible. It may be understood that, in the Aboriginal knowledge landscape, “knowledge is a function of the performance and embodiment of history” and therefore “its performative nature ensures its embeddedness in narrative” and during the process of knowledge co-creation “truth emerges like a tangent to a narrative – momentary and structure like a fiction” (18). As Western science hide their metanarrative, Yolngu science foregrounds and celebrates it, where the end result is a socially and ecologically sustainable Aboriginal knowledge practice (18). However, due to the transformative and performative nature of narratives in the Yolngu culture, not all members of the community are equally able to access the same level of meaning at once – deeper meanings are only accessible to people with greater knowledge and it is their responsibility to teach younger people the layers of meaning.

The next example is the use textual metaphor to illustrate the point that social media and mobile phone text messages may be inadequate in their approach to bridge the non-Indigenous and Indigenous knowledge landscape. According to Grounded Aboriginal Theory, Aboriginal intellectuals argue that “there is no progressive enlightenment, just working together in and with the world as we find it” (18). When a new piece of knowledge or technology is introduced into the community, they are embraced after a period of assessment and valuation “in the context of community sharing and working together” (18).

The use of textual metaphors from Senior’s research on health communication from an Aboriginal standpoint further explores this process (16).

**From Distant Shores** (Rogers 1989) in Senior (16) You look to the east as the sun sinks to the west Black clouds gathering in some distant shores Then you feel the coming of the rain, so I can feel the pain Where the south wind blows nobody knows where it goes You look to the east and the sun sinks in the west Where are all the people of this world, where are we going? Killing one another, destruction, pollution-the world has its ways Even though we tried our very best we must put it to an end Where the south wind blows nobody knows where it goes You look to the east and the sun sinks to the west.

The author of this song described the poem as an “AIDS song” that he wrote in order to “tell my people about this disease that is coming” (16). On first reading, the public health message in this song remains opaque. The meaning only becomes clear through talking about the words and the meanings embedded in them. In this song the new disease is likened to the rain at the beginning of the wet season. Although you can feel the rain coming, it is extremely difficult to predict where it will fall. As the author explained “some old people can try and stop the rain, but they get tired”.

The metaphor in the song is then extended to other global problems to which people may feel powerless to prevent at the local level. These are messages, unlike the AIDS message (essentially new material), which people did have knowledge of and the author is reminding them of the other forces of destruction which have been thrust upon them from an outside world.

The point of this story, like the bark paintings, is its multiple levels of meaning. This song is the beginning of a conversation about AIDS where the information is not embedded in the message. The story requires interpretation and discussion and what is locally called “sittin telling story”. As such, it becomes a powerful health promotion tool, which can be discussed in repeated settings, as the meanings behind the words are carefully teased out.

This type of message is very different from the standard public health text message or short video, which assumes that people will be motivated to change their behavior if they receive a succinct message containing all the information they require. The latter type of message provides information to an individual, which is an end point in itself, rather than information intended for a group and as a tool to initiate discussion over a period of time.

The challenge for health services in the context of a traditional Aboriginal nation facing modernity and globalisation is to re-think the agenda of transferring western biomedical knowledge into the Aboriginal knowledge landscape. The services may instead consider creating opportunities for discourse and embrace the contribution of Aboriginal Grounded Theory in behavior change programs despite its ultra-subjectivity and avoidance of negation and persuasion.

## Conclusion

This study sought to highlight the unintentional use of social media and mobile phones as a tool for knowledge transfer between two separate knowledge landscapes despite the technology’s ability for discourse. This is not due to the unwillingness of Indigenous health services provider to engage in a culturally sensitive and secure process online, but rather the overwhelming culture in the health services provision that focuses primarily on pharmacological eradication of disease and the alteration of lifestyle choices as a result of colonisation and marginalisation, without simultaneously addressing the social determinants of Indigenous health.

Priority was given to the use of emergent technology instead of discourse or content design, a vital aspect of communications, where the brevity of such one-off information broadcasting as absolute truth runs counter-intuitive to the metaphorical, discursive and sans negation method of Aboriginal Grounded Theory popularly known in the community as ‘sittin talking story’.

We do not discount the potential of digital technologies to engage with Aboriginal communities. Instead, we propose that we re-think how this tool is used and what engaging and educational content may look like. It is essential that messages are not considered to be an end in itself, but as the beginning of an extended conversation. Video ethnographic methods that capture interactions, discourse, performance, narratives, body language, culture, law and ceremony, shared via social media and mobile phones in a culturally relevant manner could become an essential component of the health promotion tool kit of the future.

## References

[1] Thomson DF, Peterson N. Donald Thomson in Arnhem Land. South Yarra: Currey O'Neil; 1983.

[2] Yunupingu D, Muller S (2009). ‘Cross-cultural challenges for Indigenous sea country management in Australia’.. Australasian Journal of Environmental Management.

[3] Williams NM. The Yolngu and their land: a system of land tenure and the fight for its recognition. Stanford: Stanford University Press; 1986.

[4] Clark M, May SK. Macassan history and heritage: journeys, encounters and influences’. Canberra: Australian National University Press; 2013.

[5] Cole K. The Aborigines of Arnhem Land. Adelaide: Rigby; 1979.

[6] Morphy H. Mutual conversion? The Methodist church and the Yolngu, with particular reference to Yirrkala. Adelaide: Australian National University Digital Collections; 2009.

[7] Rasmussen ML. Sex education, bodily orientation and the Northern Territory Intervention. In: Poyntz SR, Kennelly J, editors. Phenomenology of youth cultures and globalization: life worlds and surplus meaning in changing times. Routledge; 2015. p. 183-212.

[8] Senior K, Chenhall R (2008). ‘Lukumbat marawana: a changing pattern of drug use by youth in a remote Aboriginal community’.. Aust J Rural Health.

[9] Senior K, Chenhall R (2013). Health beliefs and behaviour.. Med Anthropol Q.

[10] Taylor J, Bern J, Senior K. River Town at the Millennium: a baseline profile for social impact planning in South-East Arnhem Land. CAEPR Research Monograph No. 18; 2000.

[11] Senior K, Helmer J, Chenhall R (2017). ‘As long as he’s coming home to me’: vulnerability, jealousy and violence in young people’s relationships in remote, rural and regional Australia.. Health Sociology Review.

[12] Carlson BL, Farrelly T, Frazer R, Borthwick F (2015). Mediating tragedy: Facebook, aboriginal peoples and suicide.. Australasian Journal of Information Systems.

[13] Taylor AJ (2012). Information communication technologies and new Indigenous mobilities? Insights from remote Northern Territory Communities.. Journal of Rural and Community Development.

[14] Brusse C, Gardner K, McAullay D, Dowden M (2014). Social media and mobile apps for health promotion in Australian Indigenous populations: scoping review.. J Med Internet Res.

[15] Svalastog AL, Allgaier J, Gajović S (2015). Navigating knowledge landscapes: on health, science, communication, media, and society.. Croat Med J.

[16] Senior KA. A gudbala laif? Health and well being in a remote Aboriginal community – what are the problems and where lies responsibility? (Unpublished PhD Thesis). Canberra: The Australian National University; 2003.

[17] Svalastog AL, Allgaier J, Martinelli L, Gajović S (2014). Distortion, confusion, and impasses: could a public dialogue within Knowledge Landscapes contribute to better communication and understanding of innovative knowledge?. Croat Med J.

[18] Christie M (2001). Aboriginal knowledge on the Internet.. Ngoonjook..

[19] Christie M (2005). Aboriginal knowledge traditions in digital environments.. The Australian Journal of Indigenous Education..

[20] Kariippanon KA, Garrawirtja D, Senior K, Kalfadellis P, Narayan V, McCoy B. Ethnography and filmmaking for Indigenous anti-tobacco social marketing. World Social Marketing Conference. Fuse Events; 2015.

[21] Gordon R, Gurrieri L (2014). Towards a reflexive turn: social marketing assemblages.. J Soc Mark.

[22] Nakata MN. Disciplining the savages, savaging the disciplines. Canberra: Aboriginal Studies Press; 2007.

[23] Attwood B. The making of the Aborigines. Allen & Unwin; 1989.

[24] Belk R, Kozinetz R. Videography and netnography. In: Kubacki K, Rundle-Thiele S, editors. Formative Research in Social Marketing. Singapore: Springer; 2017. p. 265-79.

[25] Kariippanon K, Senior K. Engagement and qualitative interviewing: an ethnographic study of the use of social media and mobile phones among remote indigenous youth. Available from: http://ro.uow.edu.au/cgi/viewcontent.cgi?article=4180&context=sspapers. Accessed: May 4, 2017.

[26] Robertson J, Pointing BS, Stevenson L, Clough AR (2013). “We Made the Rule, We Have to Stick to It”: towards effective management of environmental tobacco smoke in remote Australian Aboriginal Communities.. Int J Environ Res Public Health.

[27] Gajović S, Svalastog AL (2016). When communicating health-related knowledge, beware of the black holes of the knowledge landscapes geography.. Croat Med J.

